# Development of an Efficient Bacterial Consortium for the Potential Remediation of Hydrocarbons from Contaminated Sites

**DOI:** 10.3389/fmicb.2016.01092

**Published:** 2016-07-14

**Authors:** Kaustuvmani Patowary, Rupshikha Patowary, Mohan C. Kalita, Suresh Deka

**Affiliations:** ^1^Environmental Biotechnology Laboratory, Life Sciences Division, Institute of Advanced Study in Science and TechnologyGuwahati, India; ^2^Department of Biotechnology, Gauhati UniversityGuwahati, India

**Keywords:** crude oil, biosurfactant, consortium, bioremediation, PAHs, *Bacillus pumilus* KS2, *Bacillus cereus* R2

## Abstract

The intrinsic biodegradability of hydrocarbons and the distribution of proficient degrading microorganisms in the environment are very crucial for the implementation of bioremediation practices. Among others, one of the most favorable methods that can enhance the effectiveness of bioremediation of hydrocarbon-contaminated environment is the application of biosurfactant producing microbes. In the present study, the biodegradation capacities of native bacterial consortia toward total petroleum hydrocarbons (TPH) with special emphasis to poly aromatic hydrocarbons were determined. The purpose of the study was to isolate TPH degrading bacterial strains from various petroleum contaminated soil of Assam, India and develop a robust bacterial consortium for bioremediation of crude oil of this native land. From a total of 23 bacterial isolates obtained from three different hydrocarbons contaminated samples five isolates, namely KS2, PG1, PG5, R1, and R2 were selected as efficient crude oil degraders with respect to their growth on crude oil enriched samples. Isolates KS2, PG1, and R2 are biosurfactant producers and PG5, R1 are non-producers. Fourteen different consortia were designed involving both biosurfactant producing and non-producing isolates. Consortium 10, which comprises two *Bacillus* strains namely, *Bacillus pumilus* KS2 and *B. cereus* R2 (identified by 16s rRNA sequencing) has shown the best result in the desired degradation of crude oil. The consortium showed degradation up to 84.15% of TPH after 5 weeks of incubation, as revealed from gravimetric analysis. FTIR (Fourier transform infrared) and GCMS (Gas chromatography-mass spectrometer) analyses were correlated with gravimetric data which reveals that the consortium has removed a wide range of petroleum hydrocarbons in comparison with abiotic control including different aliphatic and aromatic hydrocarbons.

## Introduction

**FIGURE 1:**
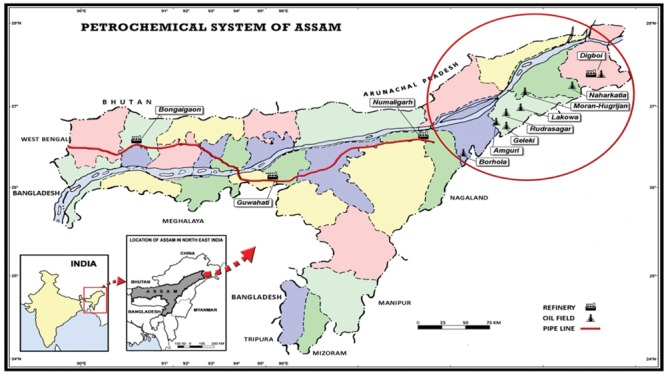
**Map of petrochemical system of state Assam, India highlighting different oilfields, refineries and pipelines**.

Assam (26.1400° N, 91.7700° E) is the oldest oil producing land from North to East India as oil drilling activity in Asia was first initiated in Digboi, Assam. The eastern part of Assam, commonly known as the upper Assam carries the main oil bearing strata extending a distance of 320 Km along the Brahmaputra valley and estimated crude oil production during 2010–2011 was 4740 TMT in Assam (**Figure 1**). This profuseness of petroleum in Assam stands both as a blessing and a curse, because unfortunately most of the crude oil drilling sites and group gathering stations (GGS; where crude oil is collected from different wells) are based at the periphery of human settlement including the agricultural belts. During the oil exploration, collection and transportation from drilling site, leakage of crude oils results in contamination of nearby agricultural fields and water bodies. Accidental and deliberate spillage and instinctive environment contamination have been a prime threat to the ecosystem and biota through the transfer of toxic substances including complex mixture of aliphatics, aromatics, nitrogen, sulfur, metals etc. into the food chain (Militon et al., 2010; Reddy et al., 2011). Due to its complex chemical nature, petroleum possesses the ability to elicit multiple types of toxic effects. It can lead to acute lethal toxicity, sub-lethal chronic toxicity, or both depending on the exposure, dosage and the organism exposed. Some of the constituents of petroleum oil have the potential to bio-accumulate within susceptible aquatic organisms and can be transferred to the subsequent level of the food chain by trophic transfer (Orisakwe et al., 2004). Furthermore, this problem is more triggered because of unsafe disposal methods owing to the associated higher cost of safe and proper disposal (Rahman et al., 2003). Thus these pernicious hydrocarbon pollutants make the development of a remediation technology essential for cleaning up polluted sites. Moreover it becomes important to adopt *in situ* remediation technique which can play a central role in removing hydrocarbon contaminants from the spilled sites before it transferred to adjacent areas where it could get accumulated (Genovese et al., 2014). Among the various strategies adopted to rehabilitate crude oil polluted sites, microbial remediation is considered as one of the potent and cost-effective technologies (Bento et al., 2005). Bacteria have long been considered as one of the predominant hydrocarbon degrading agents found in the environment, which are free living and ubiquitous (Dasgupta et al., 2013).

The success of bioremediation technologies depends on the biodegrading capabilities of native microbial populations as exogenously applied microorganisms generally fail to perform at the expected level in the foreign environment (Díaz-Ramírez et al., 2003; Venosa and Zhu, 2003). The indigenous communities which were exposed to hydrocarbons become acquainted, exhibiting selective enrichment and genetic modifications (Atlas and Bartha, 1998). The adapted microbial communities can respond to the presence of hydrocarbon pollutants within a relatively short span of time and exhibit higher biodegradation rates than communities with no history of exposure in such conditions (Atlas and Bartha, 1998). Thus, the isolation of indigenous microorganism with high oil degrading ability from a specific contaminated environment can serve as promising for the remediation of such contaminated sites. In fact it is generally considered that those microorganisms are the most active hydrocarbon degraders of that environment (Atlas and Bartha, 1998). Bioremediation of complex hydrocarbon mixture usually necessitate the cooperation of more than a single species, because an individual microorganism can generally metabolize only a limited range of hydrocarbon substrates. Therefore, conglomerations of mixed populations equipped with broad enzymatic capacities are entailed to increase the rate and extent of petroleum biodegradation further. In spite of the presence of a wide variety of hydrocarbon-degrading bacteria, growth of certain microorganisms on hydrocarbon substrates could be thwarted by numerous factors like the substrate recalcitrance and the poor solubility of hydrophobic organic compounds in aqueous systems which limit their bioavailability for biodegradation (Calvo et al., 2009; Joutey et al., 2013). Some members of the microbial community might have the ability to secrete important degradative enzymes and growth factors, whereas others can exhibit the potentiality of biosurfactant production leading to the enhanced solubilization of hydrophobic hydrocarbons for their better utilization by microbes (Ghazali et al., 2004).

Biosurfactants are surface-active amphipathic metabolites produced by certain microorganisms. Biosurfactant reduces surface tension (ST) and critical micelle dilution (CMD) in both aqueous solution and hydrocarbon mixtures, thereby facilitating the creation of micro-emulsions with the formation of micelle, in which hydrocarbons can solubilize in water or water in hydrocarbons (Banat et al., 2014). Biosurfactants are less toxic and more biodegradable than synthetic surfactants and thus considered to be more suitable for various environmental applications like hydrocarbon remediation (Antoniou et al., 2015). Accordingly inclusion of biosurfactant producing strains in a hydrocarbon degrading consortium has been considered as an interesting approach (Mnif et al., 2015). Such practice would offer the advantage of a continuous supply of biosurfactants at a low cost. Also, the application of microbes posing coexisting capacity and to degrade hydrocarbons along with the production of biosurfactants can effectively expedite the bioremediation of hydrocarbon polluted environment (Kumar et al., 2006). Moreover, hydrocarbon-degrading bacteria are well known for their ability to produce biosurfactants *in situ*, thereby facilitating their survival in hydrocarbon contaminated environments (Ganesh and Lin, 2009; Reddy et al., 2010).

Over the years, numerous studies have described the application of microbial consortia for crude oil degradation throughout the world (Rahman et al., 2002; Plaza et al., 2008; Sathishkumar et al., 2008; Bao et al., 2012). But studies on degradation of crude oil by employing indigenous bacterial consortia from this petro-chemically important geographical region are very limited (Das and Mukherjee, 2007). With relation to this, the present study had focused on the approach to elevate the level of crude oil degradation using a legitimate native bacterial consortium which amalgamates two newly isolated biosurfactant producing strains.

## Materials and Methods

### Crude Oil, Soil Samples, and Chemicals

Crude oil was obtained from Digboi Refinery, Assam, India and has been used throughout the study. Soil Samples for bacterial isolation were collected from the oilfields of Lakwa (27.01356 °N, 94.857249 °E) and Rudrasagar (26.9 °N, 94.5834 °E) situated at upper Assam and oil-contaminated garage soil obtained from Pathsala (26.4994 °N, 91.1793 °E), Assam, India. Media and chemicals of purity grade from Himedia, Merck, and Sigma has been used throughout the study.

### Microorganisms

For bacterial isolation, 1g of each hydrocarbon contaminated soil samples were inoculated in mineral salt medium (MSM) containing 2% (v/v) crude oil as carbon source for enrichment. The composition of the MSM used was as follows (g l^-1^): KCl (0.1), NaNO_3_ (4.0), K_2_HPO_4_ (1.0), KH_2_PO_4_ (0.5), CaCl_2_ (0.01), MgSO_4_⋅7H_2_O (0.5), FeSO_4_⋅7H_2_O (0.01), Yeast extract (0.1), and 10 ml of trace element solution containing (g l^-1^): CuSO_4_⋅5H_2_O (0.5), H_3_BO_3_ (0.26), (NH_4_)_6_Mo_7_O_24_⋅4H_2_O (0.06), MnSO_4_⋅7H_2_O (0.5) and ZnSO_4_⋅7H_2_O(0.7). The pH of the medium was adjusted to 7.0 ± 0.2. The conical flasks were then incubated at 35°C at 150 rpm for 7 days. After 7 days, 1 ml inoculum was added to 100 ml of fresh MSM and incubated again under similar conditions for another 7 days to decrease unwanted microbial load. Again, after 7 days 1 ml of the culture media, was used for serial dilution followed by spreading of 100 μl from 10^-4^ to 10^-6^ diluted samples on nutrient agar plates and incubation of the plates at 35°C for 24 h. Morphologically different colonies were then selected and separately streaked on nutrient agar plates so as to obtain pure culture of the bacterial isolates. The isolates were maintained in nutrient agar slant and preserved in 30% glycerol storing at -80°C incubator.

Along with these isolates, previously reported efficient hydrocarbon degrader bacterial strain *Bacillus pumilus* KS2 (GenBank accession no. KF021245; Patowary et al., 2014) was also included for further experimentations.

### Selection of Efficient Hydrocarbon Degrading Bacterial Isolates

Efficient crude oil degrading bacteria were selected depending on their growth in crude oil enriched condition. The screening was made as per the method mentioned by Rahman et al. (2002) with slight modification. At first, mother cultures of same optical density (OD_600_ = 1.0) were prepared from all the bacterial isolates in nutrient broth (NB). For the screening, 5 mL mother culture of each bacterium was inoculated into 500 mL Erlenmeyer flasks containing 100 mL of sterilized mineral medium enriched with 2% (v/v) crude oil and kept in a shaking incubator for 7 days at 35°C and 200 rpm. A set of flasks containing same composition of culture media was also maintained in same conditions as abiotic control where no inoculums were added. The bacterial growth in the medium of each flask at 0th and 7th day was estimated by taking optical density at 600 nm by UV-Vis spectrophotometer (Shimadzu UV-1800, Japan). The bacterial isolates showing maximum growth in crude oil containing media was selected for consortia development.

### Screening for Biosurfactant Producing Bacteria

Seed inoculums of the selected bacterial isolates were prepared as mentioned earlier. An amount of 5 mL mother inoculums were inoculated to 500 mL Erlenmeyer flasks containing 100 mL MSM enriched with 2% (w/v) glucose as the carbon source and incubated at 35°C with shaking at 200 rpm. Production of biosurfactant of the bacterial isolates was assayed in terms of drop collapse assay and ST reduction of the culture medium.

#### Drop Collapse Assay and Surface Tension Measurement

Drop collapse assay was performed using crude oil as hydrocarbon substrate in a slightly modified way (Bodour and Miller-Maier, 1998). As the main intent of this study was the degradation of crude oil, we used the same crude oil hydrocarbon substrate for this assay. A single drop of crude oil was set on a glass slide, following which a single drop of 48-h-grown culture broth was dropped onto the crude oil drop and drop collapse activity was observed.

Reduction of the ST of culture broths was measured after every 24 h up to 5th day of culture with the tensiometer (K11, Kruss, Germany). Culture broths were centrifuged at 10000 rpm for 10 min and filtered cell-free supernatants were used for ST determination. The isolates that could reduce ST of the culture medium below 35 mN m^-1^ were screened as efficient biosurfactant producers from the previously selected potent hydrocarbon degrader isolates.

### Development of Consortia of the Selected Bacteria

Different consortia were designed taking selected hydrocarbon degrading bacteria in the following three criteria, (i) Combination of biosurfactant producer and non-producers strains; (ii) combination of only biosurfactant producer strains; and (iii) combination of only non-biosurfactant producer strain.

### Selection of Best Consortium

Mother cultures were prepared from all of the selected bacterial isolates in NB as per the procedure mentioned above. For this selection, 5-ml seed inoculums of each consortium was inoculated into a 500-ml Erlenmeyer flask containing 100 ml of sterilized mineral medium with 2% (v/v) crude oil cultured in a shaking incubator at 35°C and 200 rpm for 1, 2, and 3 weeks. For this, mother inoculums were prepared by adding equal amount of respective bacterial culture (OD_600nm_ = 1.0) for different consortia and the final volume for inoculation was made up to 5 ml. Remaining crude oil from the culture broth after every incubation period was extracted using solvent extraction method by dichloromethane (DCM) and after solvent evaporation stored at previously weighed clean glass beaker. The amount of remaining crude oil was determined gravimetrically and thus crude oil degradation by each consortium at different incubation time was calculated. The degradation percentage of hydrocarbon was calculated following the formula proposed by Ganesh and Lin (2009), throughout the study.

Hydrocarbon degradation(%)={(Weight of residual crude oil in the abiotic control-Weight ofresidual crude oil in the test sample)Original weight of crude oil introduced}×100

### Identification of the Members of Selected Consortium

**FIGURE 2:**
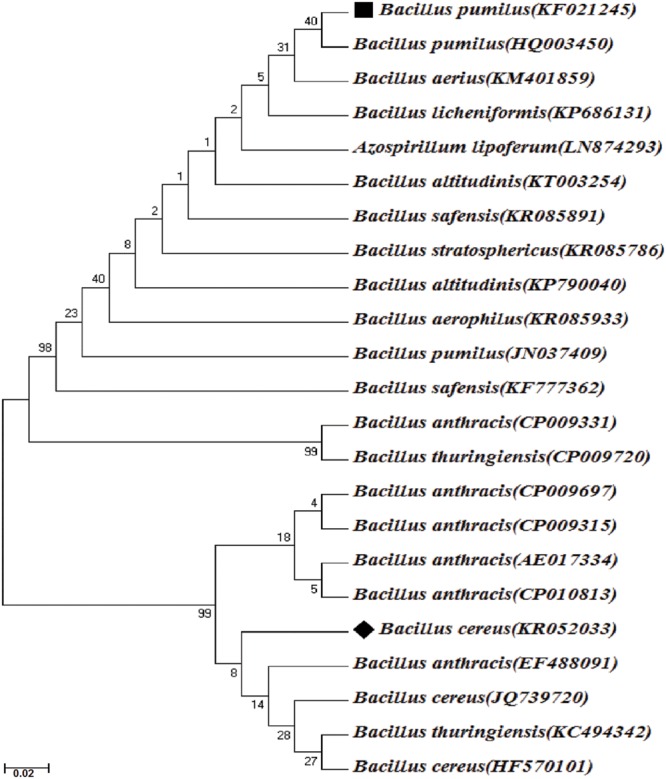
**Phylogenetic tree of *Bacillus pumilus* strain KS2 and *B. cereus* strain R2 based on 16S rDNA sequencing and their closest relatives.** Bar 0.02 nucleotide substitutions, values in parenthesis denotes GenBank accession number. ^∗^ Bootstrap values (expressed as percentages of 1000 replications) are shown at branch points.

The isolate R2 was then identified by (1) studying the morphological and physiological characteristics (Cappuccino and Sherman, 1999) (2) biochemical identification according to Bergey’s Manual of Systematic Bacteriology (Vos et al., 2009), by (3) molecular identification in which the genomic DNA of the bacteria was extracted using standard protocol. The16S rDNA was polymerase chain reaction (PCR) amplified using universal primer pair, 968F (5′-AACGCGAAGAACCTTAC -3′) and 1541R (5′-AAGGAGGTGATCCAGCCGCA -3′; White et al., 1990). PCR was performed in a 25 μl volume in thermal cycler (Mastercycler Nexus gradient, Eppendorf, Germany) with a final concentration of 1X standard buffer, 1.5 m mol l^-1^ MgCl_2_, 0.2 μ mol l^-1^ each primer, 0.2 m mol l^-1^ dNTPs and 0.25 U Taq DNA polymerase (Sigma–Aldrich, USA) and 25 ng of template DNA. The PCR reaction conditions consisted of initial denaturation at 94°C for 5 min followed by 35 cycles of denaturation at 94°C for 30 s, annealing at 60°C for 30 s, extension at 72°C for 45 s, and a final extension at 72°C for 7 min. PCR products were analyzed on 1.2% agarose gel and visualized under Bio Doc-It Imaging System (UVP, USA). PCR products were purified with GenElute^TM^ PCR Clean-Up Kit (Sigma–Aldrich, USA). PCR products were sequenced bi-directionally using an automated sequencer by Beckman coulter (GenomeLab GeXP, Genetic Analysis System, USA). 16S rDNA consensus sequence was used for Basic Local Alignment Search Tool (BLAST) analysis (Altschul et al., 1990) against nr database in the National Centre for Biotechnology Information (NCBI) GenBank^1^. Sequence data were aligned using ClustalW (Thompson et al., 1994) and phylogenetic relationship among the strains were determined by neighbor-joining method using MEGA 6 software (Tamura et al., 2013; **Figure 2**).

### Characterization of the Biosurfactants Produced by *B. pumilus* KS2 and *B. cereus* R2

Crude biosurfactant produced by biosurfactant producing strains *B. pumilus* KS2 and *B. cereus* R2 were extracted from 48 h old culture broth containing 2% (w/v) glucose as sole carbon source. Extraction was done by employing standardized solvent extraction method using ethyl acetate and subsequently purified by silica gel column chromatography (Patowary et al., 2016). Primary characterization of the biosurfactant was carried out using ninhydrin test, anthrone test, saponification test and rhamnose test following the standard methodology (Patowary et al., 2015, 2016). The ninhydrin test was carried out to determine the presence of amino acids and their polymer proteins, the anthrone test was performed to determine the presence of a carbohydrate moiety in the biosurfactant sample and the saponification test was done to detect the presence of lipid in the sample. Quantification of rhamnose sugar in the biosurfactant sample was performed by rhamnose test.

Structural characterization was done by using FTIR analysis. The column-purified biosurfactant was analyzed in NICOLET 6700 FTIR-Spectrophotometer (USA), in ATR (Attenuated total reflectance) mode considering a range of 500–4000 cm^-1^ for detection of functional groups and existing bond types.

### Degradation of Crude Oil by the Selected Consortium

Selected bacterial consortium was employed for degradation of crude oil in shake flask condition. Mother inoculums (5%) of the selected consortium were aseptically added to respective flasks containing 100 ml MSM with 2% (v/v) crude oil as sole carbon source. Mother inoculums of the selected bacterial consortium were prepared as mentioned earlier. Inoculated flasks were kept at 35°C and 200 rpm consecutively till 6th week. A set of flasks containing same composition of culture media was also maintained in same conditions as abiotic control where no inoculums were added. The optical density of the media (OD_600__nm_) was estimated to determine the growth of the consortium before extraction of residual crude oil after every weeks of culture. ST reduction was monitored after every week with the tensiometer (K11, Kruss, Germany). Culture broths were centrifuged at 10000 rpm for 10 min and filtered cell-free supernatants were used for ST determination. Residual crude oil from the culture broth after every week was extracted employing the same method described earlier. The gravimetric analysis was adopted to determine the amount of residual crude.

The degradation of various hydrocarbon fractions was analyzed in the crude oil samples extracted from the flasks containing MSM with 2% (v/v) crude oil which was inoculated with consortium 10 after 5 weeks of culture, where highest total petroleum hydrocarbon (TPH) degradation was observed in gravimetric assay. Crude oil from the abiotic control was also extracted for analysis and comparison. Extracted crude oil samples were analyzed by FTIR spectroscopy and GCMS technique to confirm the degradation efficacy of the selected consortium. For FTIR analysis the DCM extracted samples of treated crude oil and un-inoculated control were subjected to NICOLET 6700 FTIR-Spectrophotometer (USA) for analysis in ATR (Attenuated transmission resonance) mode considering a range of 500–4000 cm^-1^.

The DCM extracted samples of treated crude oil and abiotic control were analyzed though a triple quadruple Gas Chromatograph-Mass Spectrometer (GC/MS TQ8030, Shimadzu, Japan) equipped with an auto-injector (AOC 20I, GC2010, E). For the detection of various petroleum hydrocarbons the GC program was optimized and all analyses were carried out with the split ratio of 20:1. Helium was used as the carrier gas with a flow rate of 1.0 mL min^-1^, maintaining an injection temperature of 300°C. The column oven temperature was set at 60°C with a hold time of 5 min and was subsequently increased to 280°C with a ramp of 8°C min^-1^ with the final hold of 37 min. The mass spectrometric data were acquired in electron ionization mode (70 eV). The ion source temperature and interface temperature for MS were set at 230 and 310°C, respectively. The mass range (m/z) was selected as 45–600 for the entire analysis. The chromatograms were analyzed with GC-MS solution software (version 4) and the compounds identification was performed by the NIST 11 library database.

### Statistical Analysis

All of the experiments were carried out three times and studied in triplicate. Results represent the mean ± standard deviation. One-way analysis of variance (ANOVA) with the least significant difference (LSD) test was conducted to determine the significant differences in hydrocarbon degradation efficacy of the bacterial strains and different consortia at different time periods. SPSS ver.18 software (Chicago, IL, USA) was used to carry out the statistical analysis.

## Results and Discussion

### Isolation of Bacteria from Hydrocarbon Contaminated Sites

A total no of 23 (22 present isolates and 1 previous isolate) bacteria were isolated from three hydrocarbon contaminated soil samples. From Lakwa oilfield soil six different isolates were obtained namely, L1, L2, L3, L4, L5, and KS2 (previous isolate). From Rudrasagar oilfield soil isolates R1, R2, R3, R4, R5, R6, and R7 were obtained. From garage soil collected from Guwahati, 10 bacteria were isolated and coined as PG1, PG2, PG3, PG4, PG5, PG6, PG7, PG8, PG9, and PG10.

The previously isolated KS2 was identified as *B. pumilus* KS2 (Genbank accession no. gb|KF021245|; Patowary et al., 2014). Biosurfactant producer strain *B. pumilus* KS2 which was isolated from Lakwa oilfield soil, showed excellent crude oil degradation ability. Therefore, the strain was included in this revamping study for crude oil degradation. However, the strain was screened before further experimentations along with the new isolates.

### Selection of Efficient Hydrocarbon Utilizing Bacterial Strains from the Isolates

**FIGURE 3:**
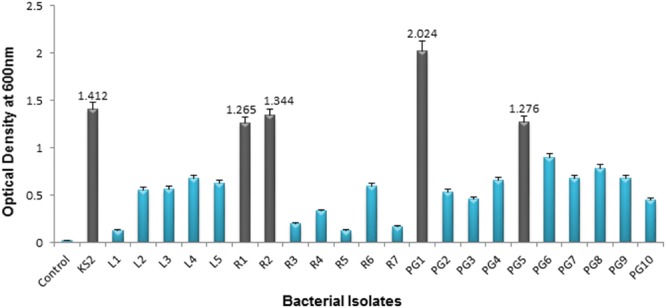
**Growth (OD_600_) of 23 hydrocarbon contaminated soil bacterial isolates on 7th day after incubation in mineral salt media supplemented with 2% (v/v) crude oil.** Error bars represent ± standard deviation (±SD).

From the total of 23 bacteria, 5 isolates, namely KS2, PG1, PG5, R1, and R2 were selected as efficient crude oil degraders based upon their significant growth (OD_600__nm_) on 2% (v/v) crude oil enriched condition (**Figure 3**). The optical density of the above mentioned isolates at 7 days culture were significantly higher than rest of the isolates. Thus, the result shows that petroleum hydrocarbons were best utilized by these isolates. Therefore, these five isolates were selected for development of consortia. Availability of nutrients like nitrogen and phosphorus plays an essential role for microbial growth in hydrocarbon contaminated environment. Again, soil temperature carries a paramount importance in remediation of hydrocarbon contaminated soil as higher temperature of soil results in higher microbial activity for degradation (Banks et al., 2000). Considering these facts we have used the MSM that contains proper amount of nitrogen and phosphorus throughout the study. Similarly as most of the bacteria prevailing in the soil system usually prefer a temperature range of 32–37°C and a nearly neutral pH for hydrocarbon degradation activity, all the experiments were carried out at 35°C maintaining a neutral pH of the medium (Ijah and Antai, 2003; Mnif et al., 2014).

The biosurfactant producing capabilities of these selected isolates were screened beforehand to facilitate appropriate combination of biosurfactant producer and non-producer isolates.

### Screening for Biosurfactant Producing Bacteria

Among the five selected isolates, culture broth of three isolates could collapse the drop of crude oil indicating the presence of biosurfactant in the culture media. The drop of crude oil was collapsed immediately within 1 min of addition of culture broth. The remaining bacterial culture broths could not collapse the drop of crude oil even after 1 min.

All three biosurfactant producer isolates that exhibits positive drop collapse assay were able to reduce the ST of the culture broth to <35 mN m^-1^ (**Table 1**). According to Joshi et al. (2008), isolates able to reduce the ST of the medium to ≤35 mN m^-1^ can be considered to be strong biosurfactant-producing microbes. Therefore, it was affirmed that three isolates namely, KS2, R2, and PG1 are efficient biosurfactant producer and isolates R1 and PG5 are biosurfactant non-producer. In the earlier report also, strain KS2 exhibited biosurfactant production when grown in glucose containing MSM (Patowary et al., 2014).

**Table 1:** Surface tension (ST) measurements of the selected bacterial isolate on glucose-containing mineral medium at different time intervals.

Selected isolates	Surface tension (mN/m) of the culture media					
	Oth h	24th h	48th h	72th h	96th h	120th h
C^a^	71.1 ± 0.30	71.0 ± 0.24	71.0 ± 0.40	69.9 ± 0.30	69.9 ± 0.23	69.8 ± 0.27
KS2^∗^	59.1 ± 0.30	30.14 ± 0.25	38.5 ± 0.30	40.2 ± 0.25	41.7 ± 0.15	43.7 ± 0.25
R1	67.2 ± 0.12	64.1 ± 0.16	63.1 ± 0.23	61.6 ± 0.32	62.5 ± 0.21	62.9 ± 0.23
R2^∗^	62.1 ± 0.25	35.1 ± 0.08	30.4 ± 0.14	46.7 ± 0.23	56.2 ± 0.32	57.8 ± 0.21
PG1^∗^	62.2 ± 0.18	30.5 ± 0.13	27.7 ± 0.32	29.3 ± 0.25	32.8 ± 0.24	41.6 ± 0.32
PG5	69.9 ± 0.23	68.6 ± 0.21	61.2 ± 0.27	59.4 ± 0.28	59.9 ± 0.18	61.2 ± 0.16

*^a^Abiotic control. ^∗^Denotes the biosurfactant producing strains. Results represented mean ± SD of five measurements.*

### Consortia Development by Selected Bacteria

A total of 14 different consortia were developed involving selected isolates in three different combinations (**Table 2**). To prepare successful microbial consortium, bacterial cultures must be compatible with each other without any antagonism among them in order to concomitantly perform all the metabolisms required for higher degradation (Sarkar et al., 2013). In a consortium for hydrocarbon remediation, it is not necessary that all the members should have the ability to produce biosurfactant (Mnif et al., 2015). The hypothesis behind the inclusion of biosurfactant non-producer isolates was that, without biosurfactant production also, a bacterium may possess efficient hydrocarbon degradation quality. The presence of biosurfactant producing strains in the consortium may lead to a better bio-availability of hydrocarbon and this might result in efficient degradation.

**Table 2:** Table representing different criteria of combination for consortia development and various consortia included in different category.

Criteria of combination	Name including the members of the consortium
(I) Combination of biosurfactant producer and non-producer isolates (considering one biosurfactant producer each time)	Consortium 1: PG1 (+) and PG5 (-)
	Consortium 2: PG1 (+) and R1 (-)
	Consortium 3: R2 (+) and PG5 (-)
	Consortium 4: R2 (+) and R1 (-)
	Consortium 5: KS2 (+) and PG5 (-)
	Consortium 6: KS2 (+) and R1 (-)
	Consortium 7: PG1 (+) + PG5 (-) and R1 (-)
	Consortium 8: KS2 (+) + PG5 (-) and R1 (-)
	Consortium 9: R2 (+) + PG5 (-) and R1 (-)

(II) Combination of only biosurfactant producer isolates	Consortium 10: KS2 (+) and R2 (+)
	Consortium 11: PG1 (+) and KS2 (+)
	Consortium 12: PG1 (+) and R2 (+)
	Consortium 13: PG1 (+) + KS2 (+) and R2 (+)

(III) Combination of only biosurfactant non-producer isolates	Consortium 14: PG5 (-) and R1 (-)

*(+) denotes biosurfactant producing bacteria and (-) denotes biosurfactant non-producing bacteria.*

### Selection of Best Consortium

From the total of 14 tested consortia, best consortium was selected based on the TPH degradation at different incubation periods. In case of consortium 10, the TPH degradation was gradually increased with time and highest degradation (68.12% of TPH) was achieved at the 3rd week of incubation. The increment in the degradation rates in successive weeks were found to be the best compared to other consortia and statistically significant (**Table 3**). Some other consortia (consortiums 3, 4, 5, and 7) exhibited good results at the end of 1st week of culture, but the degradation rates progressively stabilized in the following 2 weeks and increment in the degradation rates in successive weeks were not statistically significant. In rest of the consortia (Consortiums 1, 2, 6, 8, 9, 11, 12, 13, and 14), degradation rates at different incubation period were lesser as compared to consortium 10 and increment in degradation rates were not statistically significant. The reason behind this may be the antagonisms among the members of a specific consortium and as a result they could not accomplish the synergistic higher degradation. Consortium 10 consists of two biosurfactant producing bacteria, *B. pumilus* KS2 and isolate R2, showed a consistently increasing drift in TPH degradation at 1, 2, and 3 weeks of incubation. As both the members of this selected consortium are biosurfactant producers, it supports the fact that efficient biosurfactant producers are good petroleum hydrocarbon degrader. The degradation time, particularly the adaptation time for hydrocarbon degrading microorganisms is usually shortened in the presence of biosurfactants (Kosaric, 2001). According to Norman et al. (2002), only when the degradation process is under rate limiting condition, the surfactant can exhibit the enhancement in the process. Biosurfactants can enhance the degradation of hydrocarbons by two mechanisms, namely, by increasing substrate bioavailability for the microorganisms and by interacting with the surface of the bacterial cell to increase the hydrophobicity of the surface, thereby allowing hydrophobic substrates to associate more easily with bacterial cells (Mulligan and Gibbs, 2004). Biosurfactants increase the surface areas of insoluble compounds which leads to better mobility and bioavailability of hydrocarbons by reducing surface and interfacial tensions. Thus, biosurfactants enhance the biodegradation and removal of hydrocarbons. Therefore, the application of biosurfactant producing bacteria to a culture system loaded with hydrocarbon contaminants can be expected to amplify the biodegradation of hydrocarbon by mobilization, solubilization or emulsification (Nievas et al., 2008; Das et al., 2014). Kumari et al. (2012) reported that biosurfactant producing strains *Pseudomonas* sp. BP10 and *Rhodococcus* sp. NJ2 degraded 60.6 and 49.5% of TPH, respectively, after 30 days of incubation in minimal salt media with 2% of crude oil at their optimum conditions. In a soil reclamation study by Das and Mukherjee (2007), three biosurfactant producer strains, namely, *Pseudomonas aeruginosa* M, *P. aeruginosa* NM and *B. subtilis* DM-04 were employed in a consortium, which could degrade various hydrocarbon constituents of crude oil at a more pronounced rate (TPH level was reduced from 84 to 21 g/kg).

**Table 3:** Petroleum hydrocarbon degradation by different consortia in different time periods.

Treatment	1st Week	2nd Week	3rd Week
Consortium 1	37.39 ± 0.05^a^	39.08 ± 0.11^b^	41.32 ± 0.07^b^
Consortium 2	28.89 ± 0.12^c^	38.72 ± 0.08^d^	40.02 ± 0.05^d^
Consortium 3	46.89 ± 0.04^e^	47.59 ± 0.09^e^	49.79 ± 0.05^f^
Consortium 4	47.89 ± 0.12^g^	48.79 ± 0.08^g^	50.66 ± 0.14^h^
Consortium 5	44.48 ± 0.16^i^	47.56 ± 0.15^j^	48.51 ± 0.05^j^
Consortium 6	38.32 ± 0.12^k^	44.82 ± 0.07^l^	51.83 ± 0.11^m^
Consortium 7	48.58 ± 0.08^n^	48.69 ± 0.13^n^	49.96 ± 0.06^n^
Consortium 8	38.52 ± 0.17^o^	46.38 ± 0.05^p^	51.46 ± 0.12^q^
Consortium 9	37.35 ± 0.07^r^	42.89 ± 0.14^s^	44.48 ± 0.09^s^
Consortium 10	46.08 ± 0.08^t^	56.74 ± 0.12^u^	68.12 ± 0.15^v^
Consortium 11	40.27 ± 0.06^w^	45.52 ± 0.21^x^	47.48 ± 0.13^x^
Consortium 12	39.38 ± 0.15^y^	48.26 ± 0.11^z^	49.24 ± 0.09^z^
Consortium 13	41.56 ± 0.11^aa^	47.46 ± 0.14^ab^	48.64 ± 0.09^ab^
Consortium 14	40.35 ± 0.08^ac^	45.56 ± 0.16^ad^	51.42 ± 0.12^ae^

*Values are in percentage (%) and average of three replicates. Mean value ± SD, different letters in the same row and column indicates the significant differences.*

### Identification of the Members of Selected Consortium

Morphological and physiological characteristics of the isolate R2 showed its resemblance with *B. cereus* having smooth surfaced, creamy and flat colony morphology and Gram-positive rod shaped structure. The biochemical study also revealed the isolate to be a *B. cereus* with positive catalase test, gelatinase test, casease test, oxidase test, nitrate reduction test, and glucose fermentation test (Gibson and Gordon, 1974). The 16S rRNA gene partial sequence of strain R2 was submitted to the NCBI GenBank database under accession no. KR052033. BLAST search was conducted to compare the sequences with existing sequences and strain R2 was identified as *B. cereus* R2. Thus, the constituents of the selected consortium were *B. pumilus* KS2 and *B. cereus* R2.

### Characterization of the Biosurfactants Produced by *B. pumilus* KS2 and *B. cereus* R2

Absence of Ruhemann’s purple complex formation in the ninhydrin test, indicates the absence of amino acids or proteins in the biosurfactant samples. The formation of a blue–green color in the anthrone test for carbohydrates implies the presence of carbohydrates in the biosurfactant samples. In the saponification test, formation of foam was observed as NaOH saponified the lipids present in the biosurfactants, indicating the presence of lipids. These results reveal that the biosurfactants produced by bacterial strain *B. pumilus* KS2 and *B. cereus* R2 contain sugar and lipid molecules. In our previous study also strain *B. pumilus* KS2 showed same biochemical characteristics (Patowary et al., 2014). The rhamnose test which was performed for quantification of rhamnolipid in the biosurfactant samples, shows that 1 g L^-1^ of the crude biosurfactant produced by *B. pumilus* KS2 and *B. cereus* R2 was equivalent to 0.72 and 0.68 g L^-1^ of rhamnolipid, respectively. Thus from the above biochemical assays, it can be concluded that the biosurfactants produced by both the strains are rhamnolipid in nature.

The FTIR spectrum of column purified biosurfactant of strain *B. cereus* R2 revealed important bands at 3335, 2923, 1730, 1639, and 1025 cm^-1^ (Supplementary Figure S1). For interpretation of various functional groups present in the biosurfactant, the FTIR spectrum was compared with Pornsunthorntawee et al. (2008). Due to the presence of hydrogen bonding, the appearance of a strong and broad band of the hydroxyl group (-OH) free stretch was observed at 3335 cm^-1^. The occurrence of C-H stretching vibrations of hydrocarbon chain of alkyl (CH_2_-CH_3_) groups was confirmed by the absorption band observed at 2923 cm^-1^. Characteristic carbonyl stretching band which denotes the presence of ester compounds was found at 1730 cm^-1^. The stretching of COO^-^ group was asserted through the deformation vibration at 1639 cm^-1^. The absorption band found at 1025 cm^-1^ is the characteristics of the glycosidic bond (C-O-C) present in the molecule. Therefore, from the above discussion it can be summarized that the chemical structure of this biosurfactant is identical to those of previously reported rhamnolipid which comprises of rhamnose ring attached with long hydrocarbon chains. In case of biosurfactant obtained from strain *B. pumilus* KS2, similar bands at 3386, 2926, 1735, 1645, and 1036 cm^-1^ were found in our previous report (Patowary et al., 2014). The identical bands and bonding patterns observed in both the biosurfactant samples confirms that the biosurfactant produced by *B. pumilus* KS2 and *B. cereus* R2 are rhamnolipid in nature.

### Degradation of Crude Oil by Selected Consortium

**FIGURE 4:**
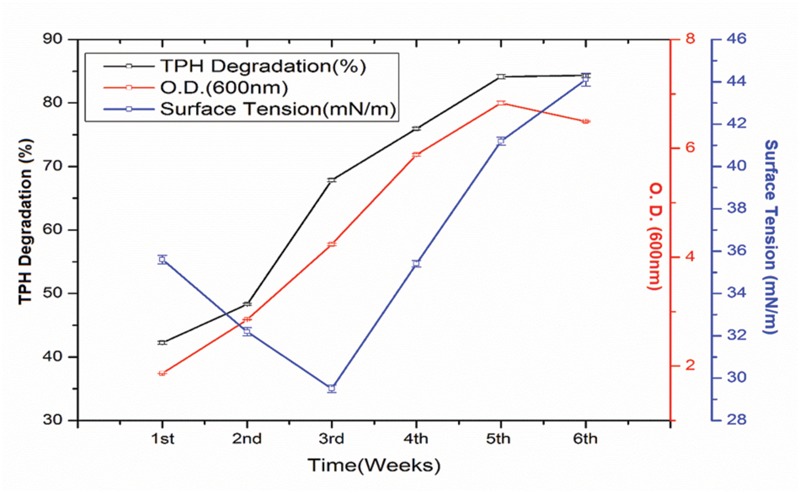
**Quantity of total petroleum hydrocarbon (TPH) degraded (%) by selected consortium at weekly intervals up to 6th week of incubation along with the optical density (growth of the consortium in the media) and ST of the culture medium.** Bars represent the ±standard deviation (±SD).

**FIGURE 5:**
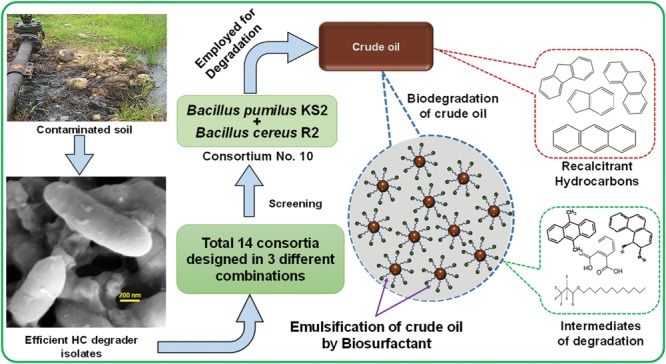
**Schematic presentation showing the activity of bacterial consortium in degrading recalcitrant petroleum hydrocarbons and simultaneous production of biosurfactant**.

The crude oil degradation rate of consortium 10 revealed that there was an increasing trend in degradation for every successive week up to the 5th week (84.15%) whereas minimum (42.24%) was recorded in the 1st week of incubation (**Figure 4**). The differences in TPH degradation values at different incubation period was found to be statistically significant, although there was no significant increase in the value after 5th week of incubation (ANOVA LSD test, *p* < 0.05). It was also observed that during the degradation process of crude oil by the consortium 10, the ST of the culture medium was reduced from 51.45 to 29.5 mN m^-1^ which signifies production of biosurfactant (**Figure 4**). The simultaneous production of biosurfactant in the culture broth with continuous crude oil degradation implies that the consortium comprising of two biosurfactant producing strains, utilizes various crude oil components as substrates for the production of biosurfactant. This in turn boosts the overall degradation process of crude oil (Antoniou et al., 2015). Existence of biosurfactant in the culture broth during crude oil degradation, indicates that the biosurfactants produced by the bacterial consortium plays a very important rate limiting role in hydrocarbon degradation by making complex hydrophobic hydrocarbons easily accessible for microbial degradation through emulsification. The mode of the microbial action in degrading recalcitrant petroleum hydrocarbons and simultaneous production of biosurfactant in this course is presented here within a schematic diagram in **Figure 5**.

In this study, the consortium of two biosurfactant producing *Bacillus* sp. showed a significantly higher degradation of crude oil compared to many recent reports which deals with the application of bacterial consortia for crude oil remediation (Rahman et al., 2002; Plaza et al., 2008; Sathishkumar et al., 2008; Mukherjee and Bordoloi, 2011; Bao et al., 2012). It is widely recognized that several gram positive bacteria belonging to the *Bacillus* genera are proficient and accomplished hydrocarbon degraders (Mnif et al., 2014). Comparably, it has been reported by Ijah and Antai (2003) that *Bacillus* sp. was the superior isolate among all the crude oil utilizing bacteria isolated from highly polluted soil samples. Among the five crude oil-degrading bacterial isolates, *Bacillus* sp. COU-28 exhibited maximum degradation ability compared to the isolates belonging to *Micrococcus varians, P. aeruginosa, Vibrio* sp., and *Alcaligenes* sp. (Ijah and Antai, 2003). It was proposed that *Bacillus* species are tolerant to high levels of hydrocarbons in soil because of their resistant endospores (Calvo et al., 2009). Thus, there is growing evidences depicting the effectiveness of application of isolates belonging to the *Bacillus* sp. for rehabilitation of oil spills. Various strains of *B. pumilus* have been reported as an efficient degrader of wide range petroleum hydrocarbons including different poly aromatic hydrocarbons (PAHs; Singh and Lin, 2008; Khanna et al., 2011; Yuliani et al., 2012). Similarly, different *B. cereus* stains has also been reported as efficient petroleum hydrocarbon degraders (Tuleva et al., 2005; Maliji et al., 2013). Although few reported strains of *B. cereus* are food-borne pathogen which are basically prevalent in aged food material and improperly cooked food (Ryan and Ray, 2004), their application in oil spill remediation shouldn’t lead to any risk of pathogenicity in human being and other livestock as it will not be directly associated with any food or fodder. Survival and activity of hydrocarbon degrading microbial community in an area where vegetation is abundant mostly depends on root exudates and their ability to provide available substrates for microbial growth (Banks et al., 2000). Generally the host plant also gets benefitted from such microbial community as majority of those bacteria acts as plant growth promoting rhizobacteria (PGPR). Therefore, inclusion of bacterial species like *B. pumilus* which possess nitrogen fixing and PGPR activity might be a very useful measure overall (Hernandez et al., 2008).

**FIGURE 6:**
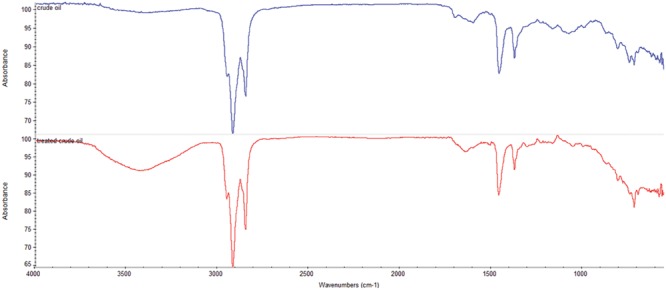
**Fourier transform infrared spectra of the DCM extracted portion of untreated control crude oil (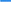) and the crude oil treated with consortium 10 for 5 weeks (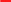)**.

Comparison of the FTIR spectra of the control (untreated crude oil sample) and the test sample (crude oil sample treated with consortium 10 for 5 weeks) distinctly demonstrates the occurrence of degradation process (**Figure 6**). In juxtaposition of the obtained FTIR spectra discloses the existence of different functional groups which unveils the degradation pattern (Pathak et al., 2015). The control and the test sample display the absorption band of CH_3_, CH_2_, and C = O stretch in similar position (2923, 2824, and 1609 cm^-1^ for control and 2915, 2854, and 1641 cm^-1^ for treated sample, respectively). However, C = O stretch found in control at 1609 cm^-1^ gives a comparatively smaller peak compared to that found in test sample at 1641 cm^-1^. Fascinatingly, the triplet of 813, 776, and 720 cm^-1^ (which indicates aromatic C = C and substituted C-H) found in degraded sample possess very lower intensity as compared to the similar triplet found in untreated sample at 816, 750, and 721 cm^-1^. Similarly, the duplets of C-C or -C-H stretch found in untreated sample at 1460 and 1380 cm^-1^ are much more prominent as compared to the same duplets found in degraded test sample at 1456 and 1374 cm^-1^. The degraded test sample gives an expanded broad peak around 3423 cm^-1^ which is absent in the control sample indicates the -OH stretching bands, which might resulted from alcohols and acids produced due to mineralization of the aliphatic and aromatic components of the crude oil during microbial activity (Bhat et al., 2011).

**FIGURE 7:**
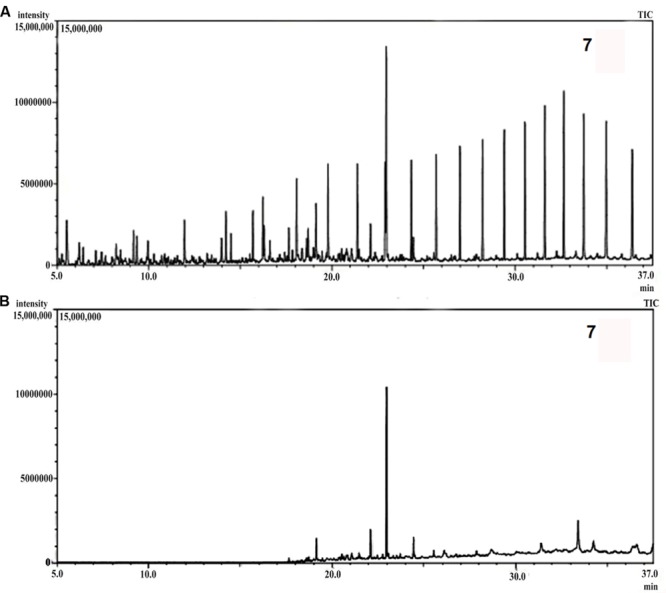
**(A)** Gas chromatography-mass spectrometer (GCMS) chromatograph of abiotic control. **(B)** GCMS chromatograph of crude oil treated for 5 weeks with consortium 10.

Gas chromatography-mass spectrometer analyses of the residual hydrocarbon extracted from consortium 10 culture was conducted after 5 weeks and compared with an abiotic control assayed under the same conditions. The obtained chromatograms are presented in **Figure 7**. From the chromatograms, it was revealed that TPH is reduced in the sample treated with the consortium as compared to the abiotic control sample. It validates the gravimetric results and suggests that the present consortium was highly effective in degrading different crude oil compounds. Microbial actions of aliphatic hydrocarbon degradation initiate by the oxidation of terminal methyl group to a primary alcohol which further converted to corresponding aldehyde through oxidation, and finally to the fatty acid derivatives. Nonetheless, in some instances, oxidation process involves both the end of alkane molecule and produces ω-hydroxy fatty acids. These ω-hydroxy fatty acids are further converted to dicarboxylic acids by β-oxidation (Coon, 2005). The secondary alcohol generated by sub-terminal oxidation of n-alkanes is converted to corresponding ketone, and then oxidized by Baeyer–Villiger monooxygenase to an ester. The resulting ester undergoes hydrolyzation by enzyme esterase generating an alcohol and a fatty acid (Singh et al., 2012). Wide ranges of alkanes (C_8_ to C_29_) including both short chain and long chain alkanes namely, n-octane (C_8_), n-undecane (C_11_), 2 bromo-dodecan (C_12_), 2,6,10-Trimethyldodecane (C_15_), n-hexadecane (C_16_), pristane (C_19_), 3-methyl-nonadecane (C_20_), didecyl eicosane (C_20_), heneicosane (C_21_), 2 methyl -tetracosane (C_24_), 3-methyl-octacosane (C_29_) and n-nonacosane (C_29_) that were present in the abiotic control sample were degraded to their derivatives or completely by the bacterial consortium. Along with other aromatic hydrocarbons, six different PAHs were detected in the untreated crude oil. From the six different PAHs detected in untreated sample, the consortium was able to completely degrade three of them, namely, 1H-Indene, 3-beta-Myristoylolean-12-en-28-ol and Anthracene. The other three PAHs, Naphthalene, Fluorene and Phenanthrene along with their various derivatives were also reduced to significantly lower concentration, but not completely after the treatment with consortium 10 (**Table 4**). Arulazhagan and Vasudevan (2009) reported that PAHs molecules namely phenanthrene and fluorene at different concentrations (5, 10, and 20 mgL^-1^) were degraded by more than 95% by the halophilic bacterial consortium they studied. Guo et al. (2005) reported a 90% degradation of phenanthrene (at 10 mg L^-1^) by a bacterial consortium isolated from mangrove sediments. Accumulation of toxic metabolites in the degradation process also leads to a decrease in the degradation rate (Vidali, 2001). In a crude oil degradation study by Malik and Ahmed (2012) the amount of anthracene and pyrene were depleted to 55.3 and 46.17%, respectively, at 24th day of treatment by bacterial consortium.

**Table 4:** Comparison of the PAHs detected in crude oil sample treated with Consortium 10 for 5 weeks and the untreated abiotic control crude oil sample.

Name of PAHs	Abiotic control crude oil	Crude oil treated for 5 weeks by Consortium 10	Degradation percentage (%)
Naphthalene	Present (7.31)	Present (2.13)	70.87
Fluorene	Present (1.23)	Present (0.47)	61.79
Phenanthrene	Present (1.56)	Present (0.64)	58.98
Anthracene	Present (1.14)	Absent	100
3-3-beta-Myristoylolean-12-en-28-ol	Present (0.34)	Absent	100
1H-Indene	Present (0.27)	Absent	100

*Values in parenthesis denotes the total area % of respective hydrocarbons.*

In the treated sample some new peaks were observed showing the generation 14 prominent degradation intermediates forming different esters and acids. Different degradation intermediates that found in the treated sample are (a)10-chlorodecyl Formic acid ester; (b) Cyclohexylmethyl oxalic acid; (c) Nonahexacontanoic acid; (d) Carbonic acid, 2-biphenyl ester; (e) octanoic acid 2-pentadecyl ester; (f) Carbonic acid, octadecyl- 2- propyl ester; (g) 9-10-Dimethylanthracene; (h) 3,4-dihydroxy-phenanthrene diol; (i) octacosanoic acid methyl ester; (j) Hexa-decanoic acid, methyl ester; (k) Pentafluoro propionic acid, dodecyl ester; (l) Phthalic acid ester; (m) Propanedioic acid, dipropyl, dimethyl ester and (n) Oxalic acid, cyclohexyl-methyl-tridecyl ester. The appearance of these new peaks resulted either from the degradation of the compounds or the synthesis of new metabolites and intermediates in the fermentation process (Seo et al., 2009; Singh et al., 2012).

## Conclusion

In this present investigation, a new bacterial consortium was developed comprising of two biosurfactant producing as well as efficient hydrocarbon degrader bacterial strain *B. pumilus* KS2 and *B. cereus* R2 isolated from crude oil contaminated soil of the oilfields of upper Assam, India. The strains of this consortium showed better compatibility with each other compared to other tested consortia and demonstrated excellent degradation of various crude oil fractions including number of recalcitrant PAHs. Therefore, this consortium could be used for the decontamination of the sites contaminated with toxic pollutants of crude oil containing PAHs. However, necessary field trials to establish the lab scale findings for cleaning up of hydrocarbon-contaminated soil by seeding this potent bacterial consortium are essential and are in progress.

## Author Contributions

KP performed all the experiments, coordinated the data analysis, and prepared the manuscript. RP contributed in the preparation of the manuscript and data analysis. MK provided the research work suggestion. SD designed the research plan and supervised the whole study.

## Conflict of Interest Statement

The authors declare that the research was conducted in the absence of any commercial or financial relationships that could be construed as a potential conflict of interest.

## Footnotes

1 www.ncbi.nlm.nih.gov
