# Supplementary material for: Development of an Efficient Bacterial Consortium for the Potential Remediation of Hydrocarbons from Contaminated Sites
Source: Front Microbiol. 2016 Jul 14;7:1092. doi: 10.3389/fmicb.2016.01092 (PMC4943938; doi:10.3389/fmicb.2016.01092)

**Supplementary Figure caption:**

**Figure S1** Fourier transform infrared spectroscopy spectra of the crude biosurfactant obtained from strain *Bacillus cereus* R2.

**Figure S1**


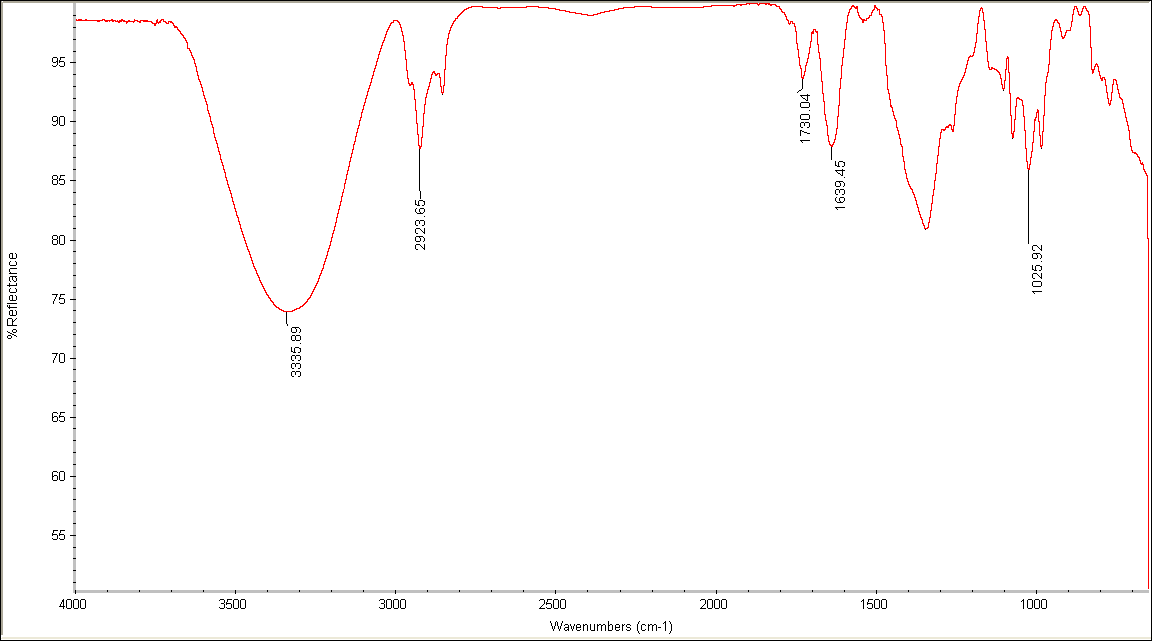

Supplement: Supplementary file 1 [file Data_Sheet_1.DOCX]
